# Macular microcirculation changes after repair of rhegmatogenous retinal detachment assessed with optical coherence tomography angiography: A systematic review and meta-analysis

**DOI:** 10.3389/fphys.2022.995353

**Published:** 2022-12-14

**Authors:** Xiaofei Chen, Wanyue Li, Xin Jin, Ying Zhang, Runpu Li, Tiecheng Liu

**Affiliations:** Senior Department of Ophthalmology, The Third Medical Center of Chinese PLA General Hospital, Beijing, China

**Keywords:** rhegmatogenous retinal detachment, macular microcirculation, foveal avascular zone, optical coherence tomography angiography, meta analysis

## Abstract

**Purpose**: The aim of the study was to investigate microcirculation changes in the macula evaluated by optical coherence tomography angiography (OCTA)in patients receiving anatomical repair after surgery for rhegmatogenous retinal detachment (RRD).

**Methods:** A literature search was conducted in PubMed, EMBASE, Web of Science and the Cochrane Library. Studies including patients with macula-on or macula-off RRD and repaired successfully through primary surgery were selected. Foveal avascular zone (FAZ) area and macular vascular density (VD) in both the superficial capillary plexus (SCP) and deep capillary plexus (DCP) were analyzed using RevMan 5.4 software.

**Results:** Twelve studies including 430 RRD eyes and 430 control eyes were selected. In eyes with macula-on RRD, FAZ area, VD in the foveal SCP and DCP, and VD in the parafoveal SCP and DCP were not altered compared with control eyes, after the retina was reattached. In eyes with macula-off RRD that was repaired successfully through surgery, FAZ area in the DCP (0.13 mm^2^, 95% CI: 0.02 to 0.25, *p* = 0.02) remained enlarged compared with control eyes. Meanwhile, VD in the foveal DCP was also significantly reduced (−3.12%, 95% CI: −6.15 to −0.09%, *p* = 0.04), even though retinal reattachment was achieved by surgery in eyes with macula-off RRD.

**Conclusion:** In patients with macula-off rhegmatogenous retinal detachment, foveal avascular zone area in the deep capillary plexuses was enlarged and vascular density in the foveal deep capillary plexus was reduced, even after the retina was successfully reattached through a primary surgery.

## Introduction

Rhegmatogenous retinal detachment is a vision-threatening disease that affects visual activity in people of working age. Although it has been reported that the retina is reattached successfully after surgery in approximately 90% of patients (Shlomit et al., 2011), the recovery of visual function is limited, especially in patients with macula-off retinal detachment. The limitation of visual recovery has been demonstrated to be related with microstructural changes of the retina in macular region, such as the disruption of ellipsoid zone and reduction of photoreceptor outer segment length (Schocket et al., 2006; Shimoda et al., 2010; Park et al., 2018).

In recent years, optical coherence tomography angiography (OCTA) has been utilized to investigate detailed changes in microcirculation in the macular area and peripapillary area in various diseases, such as diabetic retinopathy, macular degeneration and glaucoma (Kuehlewein et al., 2015; Yarmohammadi et al., 2016; Greig et al., 2020; Zhang et al., 2020). In patients with diabetic retinopathy, an increased foveal avascular zone (FAZ) area, intraretinal microvascular abnormalities, and a decrease in vascular density (VD) in macular and peripapillary areas, as detected *via* OCTA, have been found correlated with progression of diabetic retinopathy and diabetic macular edema (Sun et al., 2019; Greig et al., 2020).

In patients with macula-off rhegmatogenous retinal detachment, FAZ area and VD in the macular area evaluated by OCTA have also been investigated. Several studies have reported that FAZ area and VD in the macular area remain different from those of healthy fellow eyes after the retina was successfully reattached through surgery (Woo et al., 2018; Agarwal et al., 2019; Tsen et al., 2019). Enlarged FAZ and decreased VD have also been showed to be associated with postoperative visual activity in eyes with successful anatomical repair (Woo et al., 2018; McKay et al., 2020; Ng et al., 2020). However, in other studies, VD in the macular area of eyes receiving surgery for retinal detachment has not changed significantly (Wang et al., 2019).

This study was to investigate and meta-analyze macular microcirculation changes assessed with OCTA after successful repair for rhegmatogenous retinal detachment with or without macular involvement.

## Materials and methods

This meta-analysis was conducted in accordance with the guidelines presented by the Meta-Analysis of Observational Studies statements (Stroup et al., 2000).

### Search strategy

The databases PubMed, EMBASE, Web of Science and the Cochrane Library were searched using the terms “retinal detachment” and “Optical Coherence Tomography Angiography”, “OCT Angiography” or “OCTA” up to 07 July 2022. The detailed search query for PubMed was showed in Supplementary Table S1. Language was restricted to English.

### Study selection

Studies included should meet all of the following inclusion criteria: 1) the affected eyes had primary rhegmatogenous retinal detachment; 2) the contralateral healthy eyes or eyes of healthy people were used as controls; 3) the retina was successfully reattached after single surgery; and 4) optical coherence tomography angiography was applied to evaluate macular microcirculation. Exclusion criteria were as follows: 1) the affected eyes had any preexisting macular impairing disease, such as diabetic retinopathy, retinal vein occlusion, macular degeneration, uveitis and high myopia (axial length>26 mm); 2) the affected eyes had previous retinal surgery; 3) retinal detachment was complicated by macular hole, epi-macular membrane, submacular membrane/subfoveal fibrous band, macular scar or choroidal detachment; 4) retina was reattached after two or more surgeries; 5) internal limiting membrane was peeled in the surgery; 6) eyes with silicone oil tamponade were included and data of eyes with gas or air tamponade could not be extracted for analysis; 7) the affected eyes had any of postoperative complications including epi-macular membrane, macular edema, macular cyst and submacular fluid; 8) normal control eyes were not included; 9) children were included. The search results were reviewed by two investigators.

## Data extraction

Data from each selected study were extracted by two investigators, including first author, year of publication, location, study design, number and mean age of patients, gender, time between initial symptoms and surgery, type of surgery, time of OCTA measurement, type of OCTA device, FAZ area, VD in the foveal area, and VD in the parafoveal area. If OCTA measurements were taken at different times after surgery, data containing sufficient information at the last time of follow up were collected. FAZ area was defined as the area inside the central border of the capillary network. VD was defined as the percentage of area occupied by vessels in a defined region. FAZ area and VD both in the superficial capillary plexus (SCP) and in the deep capillary plexus (DCP) were extracted. In studies in which the layer for measurement was not mentioned, the data were analyzed as in the SCP group in our study. Discrepancies were addressed through discussion.

### Quality assessment

Method quality of the selected studies was evaluated according to the Newcastle-Ottawa Scale (NOS) (Stang, 2010). Scores ranging from 1 to 9 were applied to assess the selection criteria of subjects, comparability between controls and cases, and measurement values of each study. This procedure was completed by two reviewers and disagreements were discussed to achieve consensus. Publication bias was evaluated by Egger’s test and Begg’s test.

### Statistical analysis

Data analysis was performed with Cochrane Collaboration’s Review Manager Software (RevMan 5.4, Cochrane Collaboration, Oxford, United Kingdom). Means and standard deviations of the area of foveal avascular zone and vessel density were calculated as continuous variables to obtain the weighted mean difference. Heterogeneity among the selected studies was tested by Chi-square test and Higgins I^2^ test. If the heterogeneity was not significant (*p* > 0.10, I^2^<50%), the fixed-effect analysis model was used. Otherwise, the random-effect model was applied. A *p* value < 0.05 was considered significant for all data analysis.

## Results

### Characteristics and quality assessments

**FIGURE 1:**
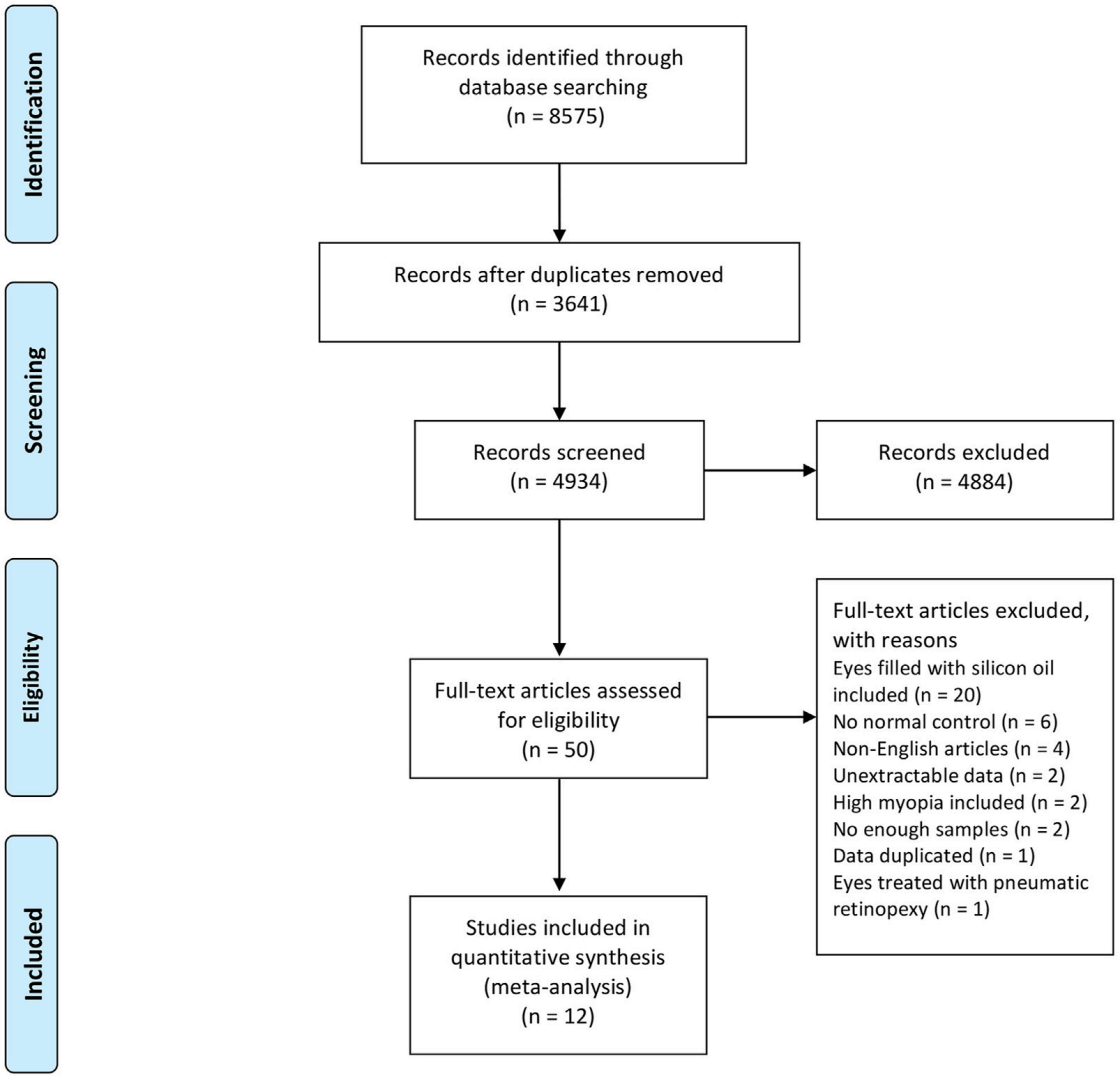
Flow chart of studies selection.

Twelve studies including 430 RRD eyes and 430 control eyes were selected in this study (Agarwal et al., 2019; Woo et al., 2018; McKay et al., 2020; Ng et al., 2020; Wang et al., 2019; Hong et al., 2020; Bonfiglio et al., 2020; Barca et al., 2020; Christou et al., 2021; Liu et al., 2021; D'Aloisio et al., 2022; Chatziralli et al., 2022), as indicated in Table 1. The search strategy for Pubmed is showed in Supplementary Table S1. The selection process is showed in Figure 1. As showed in Table 1, two studies selected macular-on RRD, six studies collected macula-off RRD, and the remaining four studies included both macula-on and macula-off RRD. In the study by D'Aloisio et al., part of the macula was detached before surgery in 6 RRD patients who received treatment within 24 h of onset (D'Aloisio et al., 2022). This group of eyes was considered as macular-on RRD for analysis in our study. Data were collected and analyzed separately in macula-on retinal detachment and macula-off retinal detachment. Patients with RRD received pars plana vitrectomy (PPV) with gas or air tamponade or scleral buckling (SB) in selected studies. One study was exclude since patients with RRD were treated with pneumatic retinopexy (Kaderli et al., 2021). Since silicone oil tamponade has been reported to be related to changes of macular microcirculation (Ma et al., 2020; Lee and Park, 2021), eyes with silicone oil tamponade were exclude in this study. The duration between symptoms and surgery was less than 14 days in most studies. However, in one study (Agarwal et al., 2019), the duration time was 1.76 ± 2.84 months (10 days–6 months), which was converted to the day unit in Table 1. The time that from surgery to OCTA measurement was at least 1 month in all studies, as showed in Table 1. As indicated in the selected studies, the foveal area was defined as a central circular region within a diameter of 1–1.5 mm, and the parafoveal area was defined as a circular annulus region between 1 and 2.5–3 mm in diameter. VD in the foveal area and the parafoveal area were analyzed separately. In the study of McKay et al. (McKay et al., 2020), VD was evaluated in a central area of 3 × 3 mm, which was not included for analysis in our study. The NOS score of each selected study is also showed in Table 1, and detailed information is showed in Supplementary Table S2.

**TABLE 1:** The characteristics of included studies.

Study	Location	Design	RRD eyes	Control eyes	Age (years)	Gender (F/M)	Status of macula		Duration between symptoms and surgery		Surgery		Time of OCTA	Device	Quality
							On	Off	On	Off	SB	PPV(T)			
Agarwal 2018	India	prospective	19	19*	40.21 ± 16.81	4/15	0	19	N	52.8 ± 85.2d	8	11 (C3F8)	3 m	Optovue	6
Woo 2018	Korea	retrospective	34	34	55.4 ± 11.1	14/20	15	19	NA	3.21 ± 2.25d	0	34 (C3F8)	<2 m	Topcon	7
Bonfiglio 2019	Italy	retrospective	93	93	62 ± 8	41/52	56	37	2.2 ± 1.1d	3.6 ± 1.3d	0	93 (SF6)	17.2 ± 5.6 m	Optovue	7
Wang 2019	China	retrospective	14	14	56.0 (42–73)	6/8	0	14	N	4.21 (3–7) d	0	14 (Air)	12 w	Optovue	7
Barca 2020	Italy	prospective	33	33	60.26 ± 11.52	13/20	15	18	1.48 ± 0.67 d		15	18 (Air)	6 m	Optovue	8
Hong 2020	Korea	retrospective	31	31	53.3 ± 14.4	8/23	11	20	6.4 ± 5.5d	7.7 ± 6.5d	0	31 (C3F8)	6 m	Topcon	8
McKay 2020	United States	retrospective	17	17	57 ± 11	6/11	0	17	N	3 ± 2d	0	17 (Gas)	4 ± 4 m	Optovue	7
Ng 2020	Netherlands	prospective	47	47	60 ± 9	11/36	0	47	N	5 (3–7)d	4	43 (C3F8 or SF6)	12 m	Heidelberg	7
Christou 2021	Greece	retrospective	23	23	62.7 ± 8.18	5/18	0	23	N	11.3 ± 8.4d	0	23 (C2F6)	12 w	Zeiss	8
Liu 2021	China	retrospective	16	16	55.5 ± 9.68	12/4	16	0	18.2 ± 15.9d	N	0	16 (C3F8)	36.3 ± 3.7 m	Optovue	8
Chatziralli 2022	Greece	prospective	89	89	67.9 ± 5.7	36/53	0	89	N	≤15d	0	89 (C3F8 or SF6)	5 w	Optovue	8
D'Aloisio 2022	Italy	prospective	14	14	52.6 ± 15.2	6/8	14	0	<24 h	N	0	14 (C3F8 or SF6)	3 m	Canon	8

*Eyes of healthy subjects were used as control in this study while the fellow eyes used as control in other studies. RRD, rhegmatogenous retinal detachment; SB, scleral buckling; PPV, pars plana vitrectomy, m, months, d, days, h, hours, NA, not available N none.

### Foveal avascular zone area

**FIGURE 2:**
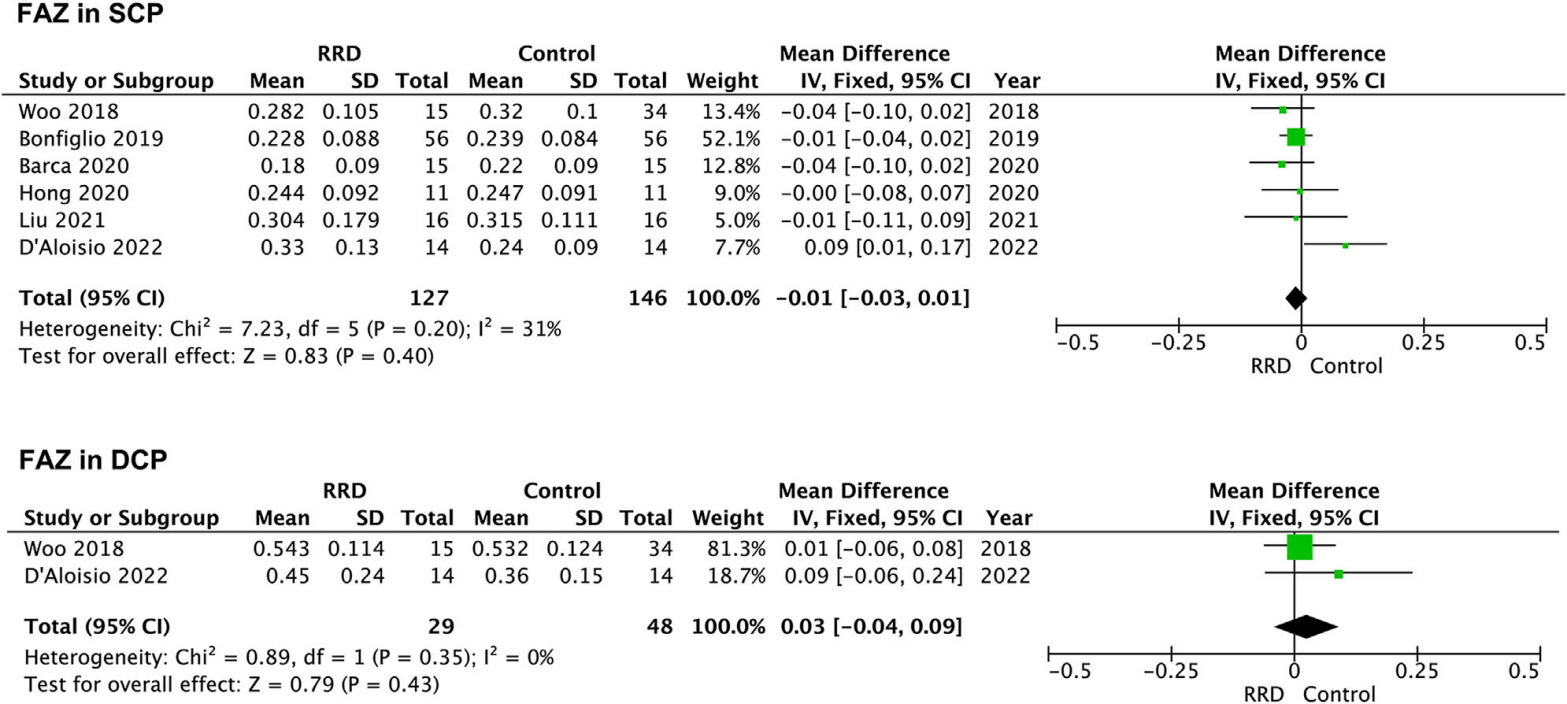
Forest plots of changes in FAZ area in eyes with Macula-on RRD. FAZ, foveal avascular zone; SCP, superficial capillary plexus; DCP, deep capillary plexus, RRD, rhegmatogenous retinal detachment.

**FIGURE 3:**
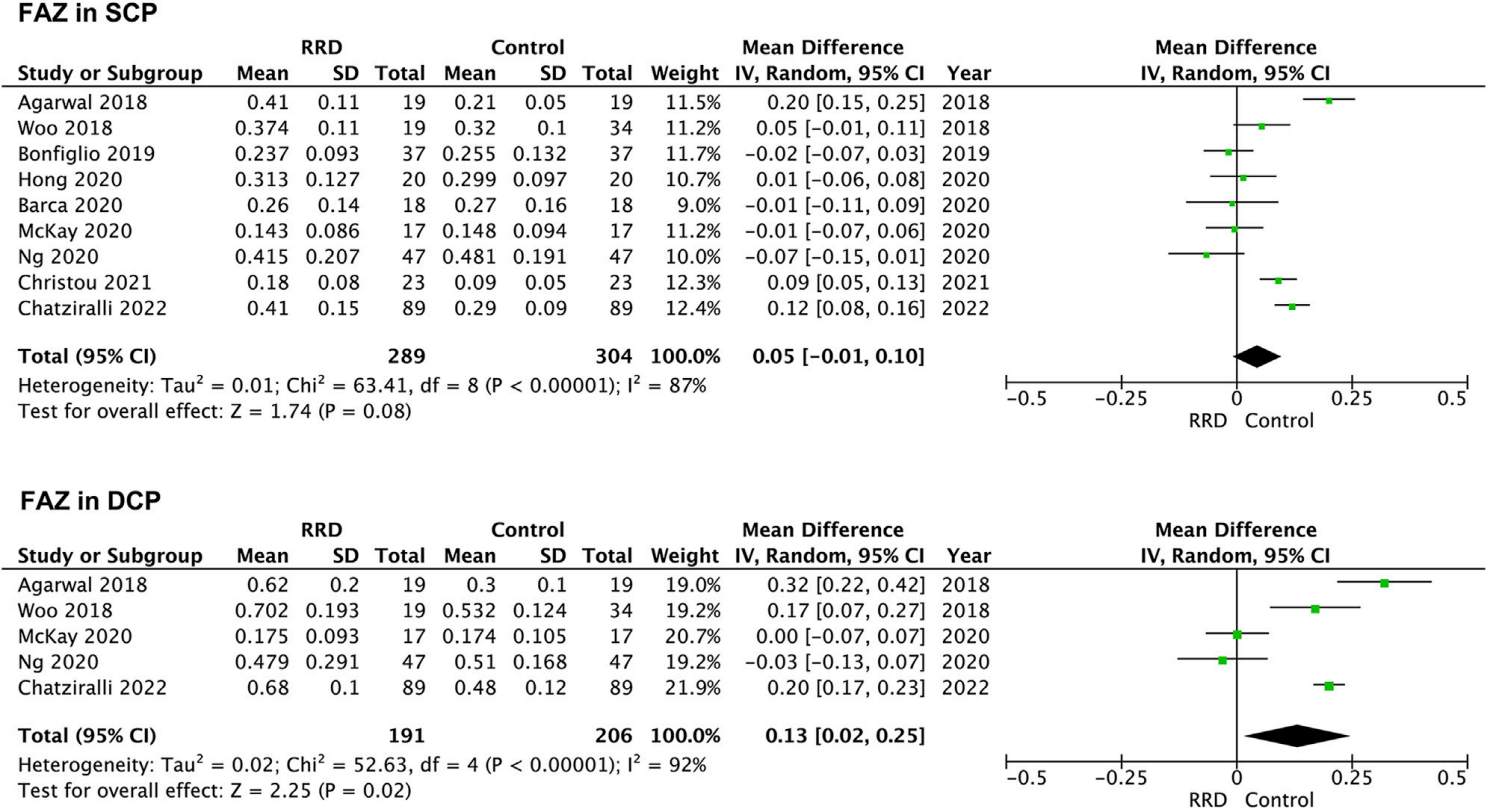
Forest plots of changes in FAZ area in eyes with Macula-off RRD. FAZ, foveal avascular zone; SCP, superficial capillary plexus; DCP, deep capillary plexus, RRD, rhegmatogenous retinal detachment.

Seven studies were included for analyzing FAZ area of macula-on RRD. As showed in Figure 2, FAZ area in the SCP and in the DCP of macula-on RRD was not different from that of control eyes after surgery (−0.01 mm^2^, 95% CI: −0.03 to 0.01, *p* = 0.40; 0.03 mm^2^, 95% CI: −0.04 to 0.09, *p* = 0.43). Nine studies collecting 289 eyes with RRD and 304 control eyes were selected for analyzing FAZ area in the SCP of macula-off RRD. FAZ area in the SCP was not changed after treatment (0.05 mm^2^, 95% CI: −0.01 to 0.10, *p* = 0.08). To calculate FAZ area in the DCP of macula-off RRD, five studies including 191 eyes in the RRD group and 206 eyes in the control group were collected. As indicated in Figure 3, FAZ area in the DCP was significantly increased even after the retina was reattached successfully through surgery in macula-off RRD eyes compared with that of control eyes (0.13 mm^2^, 95% CI: 0.02 to 0.25, *p* = 0.02).

### Vascular density in the foveal area

**FIGURE 4:**
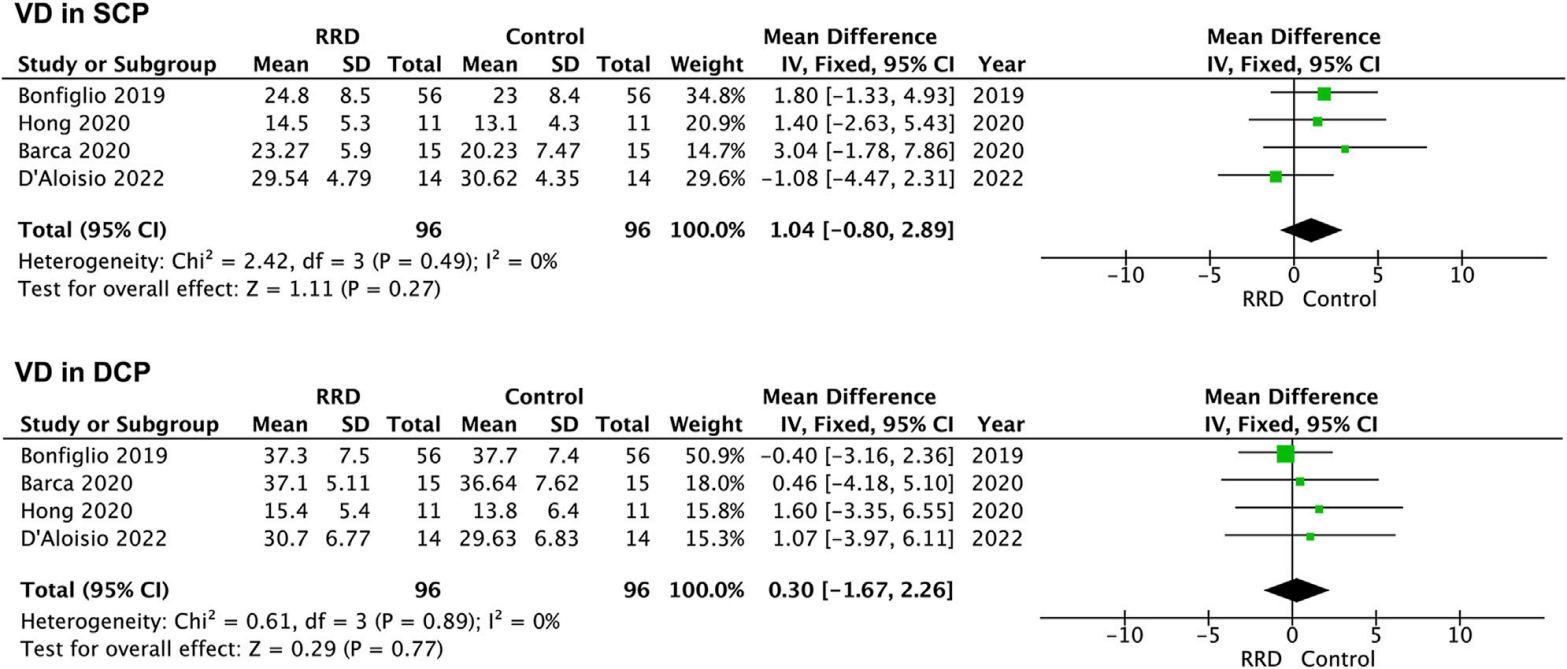
Forest plots of changes in foveal VD in eyes with Macula-on RRD. VD, vascular density, SCP, superficial capillary plexus; DCP, deep capillary plexus, RRD, rhegmatogenous retinal detachment.

**FIGURE 5:**
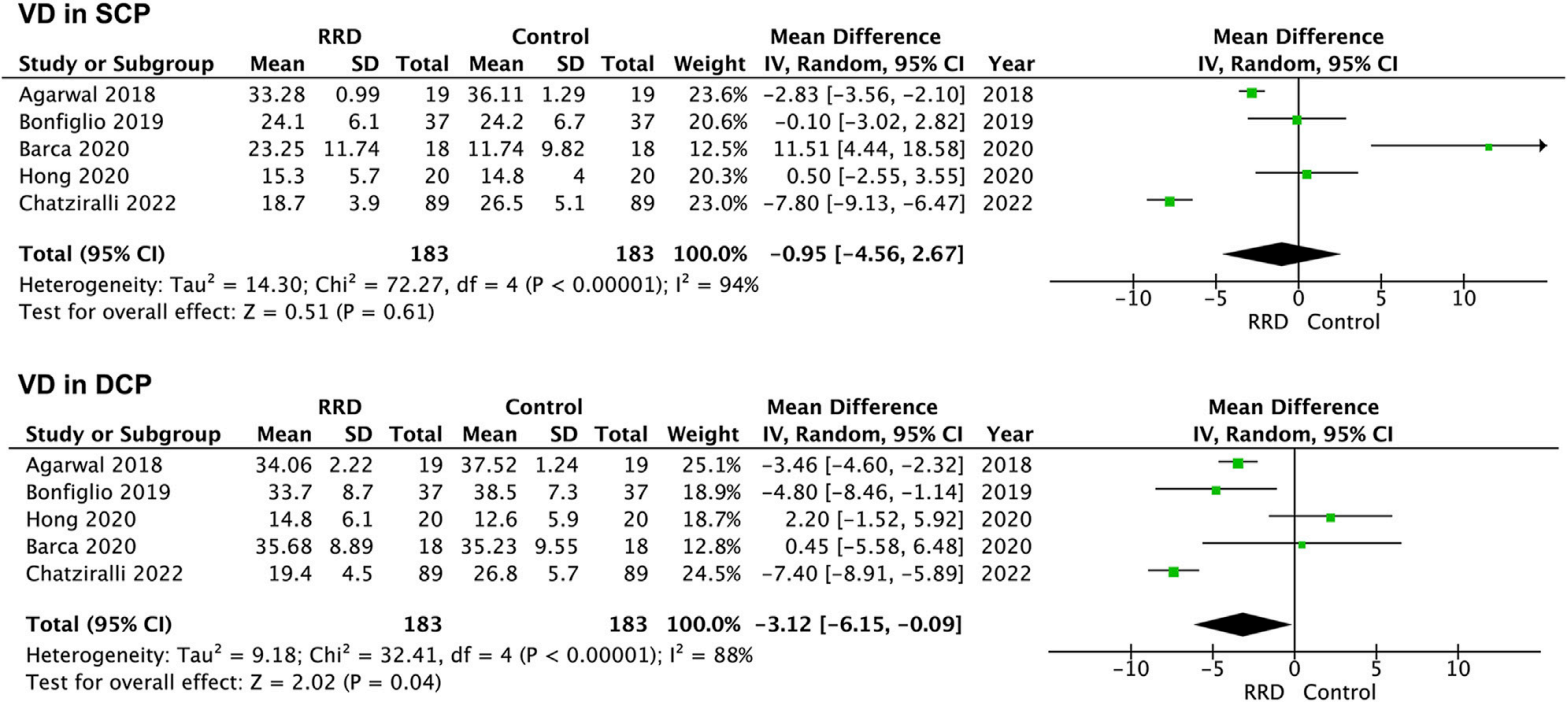
Forest plots of changes in foveal VD in eyes with Macula-off RRD. VD, vascular density, SCP, superficial capillary plexus; DCP, deep capillary plexus, RRD, rhegmatogenous retinal detachment.

In macula-on RRD, VD in the SCP and in the DCP of the foveal area was not changed compared with that of control eyes after surgery (1.04%, 95% CI: −0.80%–2.89%, *p* = 0.27; −0.30%, 95% CI: −1.67%–2.26%, *p* = 0.77, Figure 4). A total of 183 eyes with RRD and 183 control eyes in five studies were analyzed for VD changes in the foveal area of macula-off RRD. As demonstrated in Figure 5, no change in VD in the SCP was observed when compared with that of controls (−0.95%, 95% CI: −4.56 to 2.67%, *p* = 0.61). However, VD in the DCP of the foveal area was significantly reduced in macula-off RRD (−3.12%, 95% CI: −6.15% to −0.09%, *p* = 0.04), even though retinal detachment was repaired successfully after surgery.

### Vascular density in the parafoveal area

**FIGURE 6:**
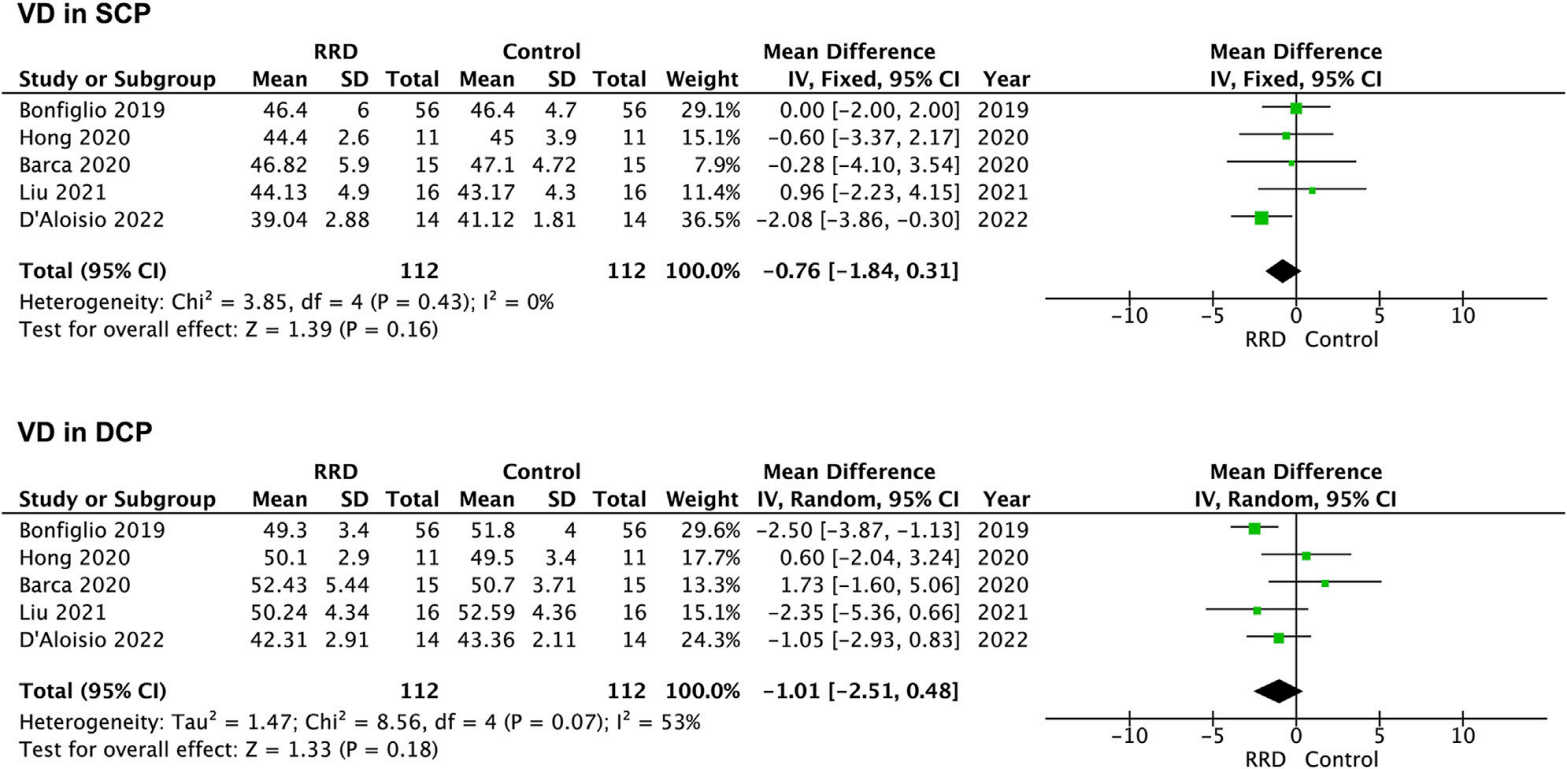
Forest plots of changes in parafoveal VD in eyes with Macula-on RRD. VD, vascular density, SCP, superficial capillary plexus; DCP, deep capillary plexus, RRD, rhegmatogenous retinal detachment.

**FIGURE 7:**
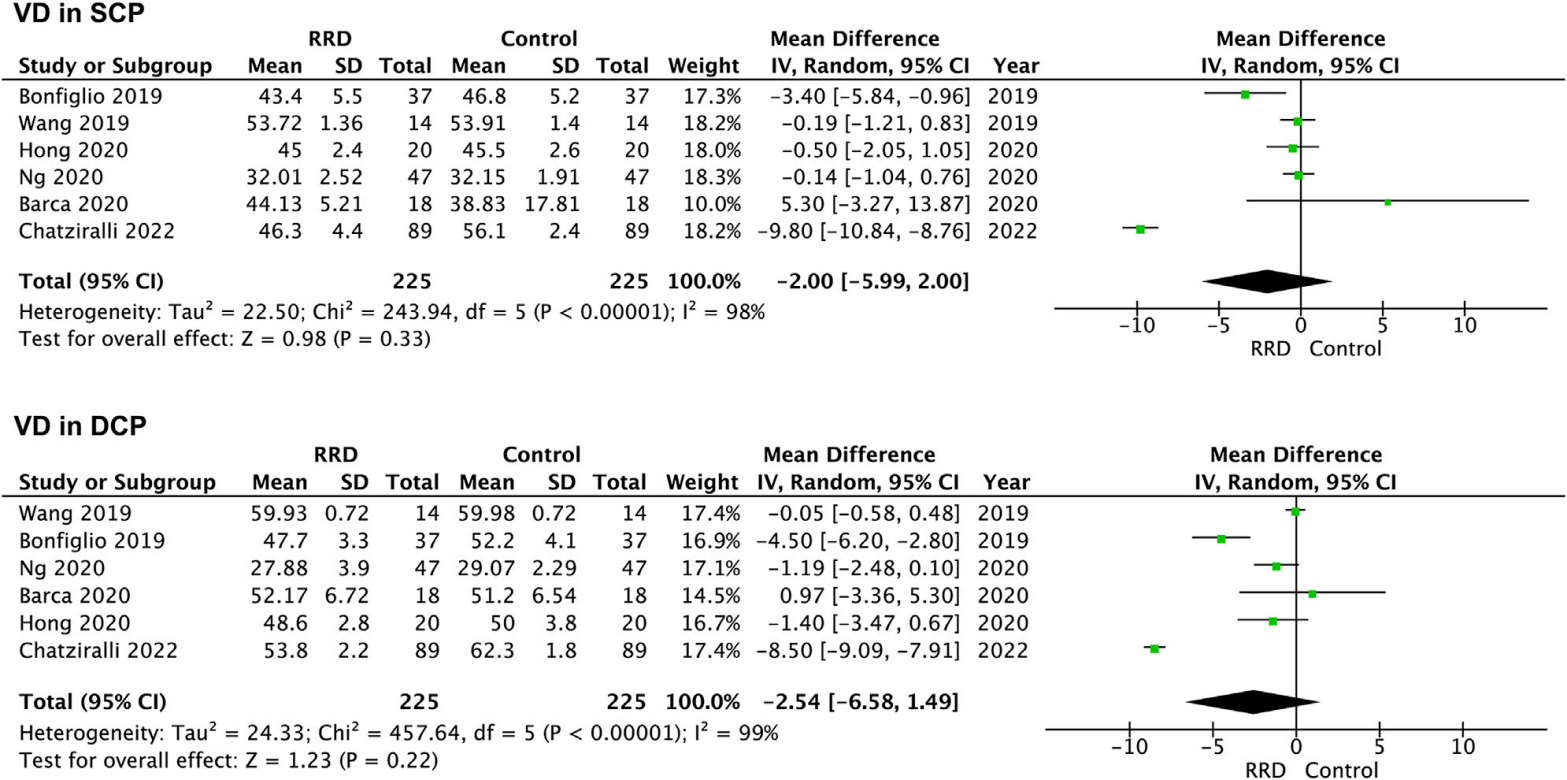
Forest plots of changes in parafoveal VD in eyes with Macula-off RRD. VD, vascular density, SCP, superficial capillary plexus; DCP, deep capillary plexus, RRD, rhegmatogenous retinal detachment.

VD in the parafoveal area was also analyzed in patients with RRD. No difference was detected in VD in the SCP or the DCP between eyes with macula-on RRD and control eyes (−0.76%, 95% CI: −1.84 to 0.31%, *p* = 0.16; −1.01%, 95% CI: −2.51 to −0.48%, *p* = 0.18, Figure 6). In patients with macula-off RRD, 225 eyes in the RRD group and 225 eyes in the control group from six studies were summarized for calculation. As showed in Figure 7 VD in the SCP and the DCP was not changed after retina was reattached (−2.00%, 95% CI: −5.99 to 2.00%, *p* = 0.33; −2.54%, 95% CI: −6.58% to 1.49%, *p* = 0.22).

### Vascular density of the choriocapillaris

**FIGURE 8:**
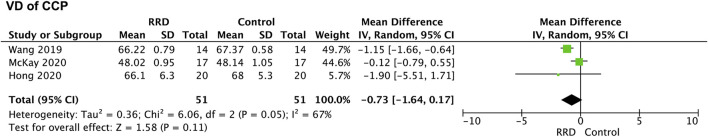
Forest plots of changes in VD of the choriocpillaris in eyes with macula-off RRD. VD, vascular density; RRD, rhegmatogenous retinal detachment.

VD of the choriocapillaris was evaluated in four studies. Three studies were selected for meta-analysis, because VD of the choriocapillaris was measured in an area of 8 mm × 8 mm while the measurement was performed within an area of 3 mm × 3 mm in other studies. VD of the choriocpillaris in eyes with macula-off RRD was analyzed. As showed in Figure 8, VD of the choriocpillaris was not altered after retinal reattachment (−0.73%, 95% CI: −1.64% to 0.17%, *p* = 0.11).

### Publication bias

Publication bias was assessed by Egger’s test and Begg’s test. No publication bias was revealed in FAZ area, VD in the foveal and the parafoveal areas of eyes with macular-off RRD. However, publication bias was found in VD in the DCP of the foveal area in eyes with macular-on RRD by Egger’s test (*p* = 0.04) but not by Begg’s test. No significant publication bias was observed in other measurements in eyes with macular-on RRD. Funnel plots of studies are showed in Supplementary Figure S1.

## Discussion

This study indicated that FAZ area in the DCP of eyes with macula-off RRD remained enlarged when the retina was reattached successfully after a single surgery, as compared with control eyes. Meanwhile, VD in the DCP of the foveal area in eyes with macula-off RRD also remained diminished after anatomical repair.

It has been reported that blood flow in the macular area was not different between eyes that received SB and those that received PPV and gas tamponade for retinal detachment (Sato et al., 2007). Other studies indicated that macular microcirculation was changed after silicone oil tamponade, including narrowing of arterioles and reduced blood flow (Agnieszka et al., 2011; Roohipoor et al., 2020). Accordingly, patients who received SB or PPV with gas tamponade, but not silicone oil tamponade, were selected in this study.

It has been found that capillaries in the DCP are more vulnerable to retinal and systemic diseases, such as retinal vein occlusion, diabetic macular edema, hypertension and systemic lupus erythematosus (Adhi et al., 2016; Lee et al., 2016; Chua et al., 2019; Arfeen et al., 2020). The mechanism is not clear. In the SCP of normal eyes, capillaries were located between arterioles and venules, forming an interconnected plexus. In the DCP, capillaries were arranged as polygonal units and were speculated to drain into the superficial venules (Bonnin et al., 2015). Moreover, as a single monolayer of the capillary plexus, VD in the DCP has been found to be lower than that in SCP (Lavia et al., 2019). The difference in structural pattern may be associated with unbalanced vulnerability to ischemia between the SCP and the DCP.

Decreased visual activity has been reported to be related to capillary loss in the DCP in type 1 diabetes without macular edema (Dupas et al., 2018). Furthermore, nonperfusion in the DCP has been proved to be correlated with photoreceptor disruption, which suggests that blood flow in the DCP is critical to the survival and function of photoreceptors (Scarinci et al., 2016).

In patients with macula-off retinal detachment, Woo et al. have reported that FAZ area in the SCP and the DCP are negatively associated with postoperative best-corrected visual acuity (BCVA) (Woo et al., 2018). In the study by Bonfiglio et al., BCVA has been proved to be related not only to FAZ area but also to VD in the foveal SCP and the parafoveal DCP (Bonfiglio et al., 2020). In McKay’s investigation, VD in the DCP, but not VD in the SCP and FAZ area, was found to be changed and correlated with BCVA (McKay et al., 2020). Ng et al. observed that a smaller FAZ area in the DCP was associated with better postoperative BCVA (Ng et al., 2020).

The pathogenetic mechanism is not clear. Deep capillaries are located in the inner nuclear layer. The avascular outer retinal layers are supplied by diffusion of oxygen and nourishment from the choriocapillaris (Tasman and Jaeger, 1991). However, it has also been found that 10%–15% of oxygen supplied to photoreceptors comes from retinal circulation (Birol et al., 2007), which indicates capillaries in the DCP may play an important role. In addition, capillaries in the DCP have also been considered important to provide oxygen and nutrition to synapses formed between photoreceptors and bipolar cells (Provis, 2001; Masland, 2012), which are therefore important for visual signal transmission. Usui et al. have speculated that vasculature, glia and neurons within retinal neurovascular units are highly interdependent and that their interactions are vital to maintain normal metabolism. Loss of either or both retinal interneurons and vasculature may lead to photoreceptor dysfunction (Usui et al., 2015). Inflammatory cytokines, such as tumor necrosis factor α (TNF-α) and monocyte chemotactic protein-1 (MCP-1), may take part in the process (Nakazawa et al., 2006; Nakazawa et al., 2011).

Some limitations should be mentioned in this meta-analysis. First, the sample size of most selected studies was small. Second, the duration between symptoms and surgery, type of surgery, time and device of OCTA examination were different among the included studies, which contributed to the heterogeneity. The consistency of measurements among different OCTA devices has been investigated. It has been reported that FAZ area and VD in the SCP show good repeatability, while VD in the DCP dose not (Anegondi et al., 2018). In another study, FAZ area measurements were consistent across different devices, while differences in VD measurements were observed (Lu et al., 2021). Subgroup analysis could be performed when more studies are collected. Third, variations in software, layer segmentation and area selection during OCTA measurement also added to the heterogeneity. The SCP and the DCP were segmented automatically in most studies. The SCP was located from 3 μm under the internal limiting membrane (ILM) to 15 μm under the inner plexiform layer (IPL), and the DCP extended from 15 to 70 μm below the IPL. In the study by Liu et al., however, SCP was located between the ILM and 10 μm above the IPL and the inner nuclear layer (IPL-INL) junction, while the DCP was located between 10 μm above the IPL-INL junction and 10 μm below the outer plexiform layer and outer nuclear layer junction. Finally, the correlation between visual activity and FAZ or VD were not analyzed because of insufficient data in the selected studies.

In conclusion, in eyes with macula-off rhegmatogenous retinal detachment, FAZ area in the DCP was enlarged and VD in foveal DCP was reduced as compared with normal control eyes, even after successful anatomical repair through primary surgery. Multicenter studies with larger sample sizes are needed to evaluate changes in macular microcirculation in rhegmatogenous retinal detachment and its association with vision recovery after surgery.

## Data Availability

The original contributions presented in the study are included in the article/Supplementary Material, further inquiries can be directed to the corresponding author.

## Supplementary material

SUPPLEMENTARY FIGURE S1 Funnel plots of studies. **(A)** , FAZ area in SCP of eyes with Macula-on RRD; **(B)**, FAZ area in DCP of eyes with Macula-on RRD; **(C)**, FAZ area in SCP of eyes with Macula-off RRD; **(D)**, FAZ area in DCP of eyes with Macula-off RRD; **(E)**, Foveal VD in SCP of eyes with Macula-on RRD; **(F)**, Foveal VD in DCP of eyes with Macula-on RRD; **(G)**, Foveal VD in SCP of eyes with Macula-off RRD; **(H)**, Foveal VD in DCP of eyes with Macula-off RRD; **(I)**, Parafoveal VD in SCP of eyes with Macula-on RRD; **(J)**, Parafoveal VD in DCP of eyes with Macula-on RRD; **(K)**, Parafoveal VD in SCP of eyes with Macula-off RRD; **(L)**, Parafoveal VD in DCP of eyes with Macula-off RRD. FAZ, foveal avascular zone; SCP, superficial capillary plexus; DCP, deep capillary plexus, RRD, rhegmatogenous retinal detachment; VD, vascular density.
