# Supplementary material for: Macular microcirculation changes after repair of rhegmatogenous retinal detachment assessed with optical coherence tomography angiography: A systematic review and meta-analysis
Source: Front Physiol. 2022 Dec 14;13:995353. doi: 10.3389/fphys.2022.995353 (PMC9795227; doi:10.3389/fphys.2022.995353)
Supplement: Supplementary file 1 [file Table1.DOCX]

**Table S1** Search Strategy for Pubmed

| Search number | Query | Sort By | Filters | Results |
| --- | --- | --- | --- | --- |
| 1 | ((((((((Optical Coherence Tomography Angiography) OR (Optical Coherence Tomographic Angiography)) OR (OCTA)) OR (OCT Angiography)) OR (angio-OCT)) OR (OCT-A)) OR (OCT-angiography)) OR (OCT-angio)) OR (OCT-angiographie) | Most Recent |  | 54,856 |
| 2 | (retinal detachment) OR (retinal detachment[MeSH Terms]) | Most Recent |  | 30,872 |
| 3 | (((((((((Optical Coherence Tomography Angiography) OR (Optical Coherence Tomographic Angiography)) OR (OCTA)) OR (OCT Angiography)) OR (angio-OCT)) OR (OCT-A)) OR (OCT-angiography)) OR (OCT-angio)) OR (OCT-angiographie)) AND ((retinal detachment) OR (retinal detachment[MeSH Terms])) | Most Recent |  | 2,067 |
