# Supplementary figures and images for: Macular microcirculation changes after repair of rhegmatogenous retinal detachment assessed with optical coherence tomography angiography: A systematic review and meta-analysis
Source: Front Physiol. 2022 Dec 14;13:995353. doi: 10.3389/fphys.2022.995353 (PMC9795227; doi:10.3389/fphys.2022.995353)

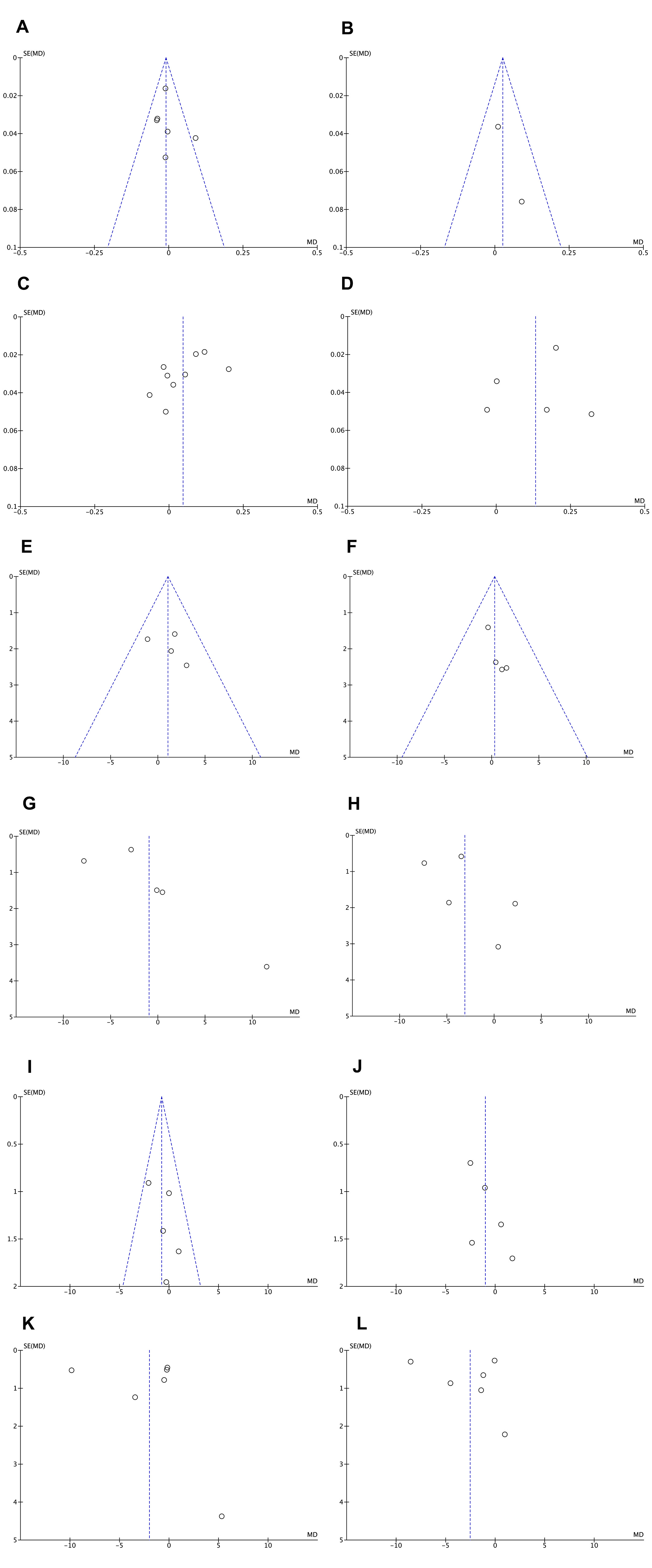

Supplement: Supplementary file 2 [file Image1.JPEG]
