# Supplementary material for: Macular microcirculation changes after repair of rhegmatogenous retinal detachment assessed with optical coherence tomography angiography: A systematic review and meta-analysis
Source: Front Physiol. 2022 Dec 14;13:995353. doi: 10.3389/fphys.2022.995353 (PMC9795227; doi:10.3389/fphys.2022.995353)
Supplement: Supplementary file 3 [file Table2.docx]

Table S2. Quality Assessment with Newcastle-Ottawa Scale

| Study | Selection | Comparability | Outcome | Quality Score |
| --- | --- | --- | --- | --- |
| Agarwal 2018 | 3 | 1 | 2 | 6 |
| Woo 2018 | 3 | 2 | 2 | 7 |
| Bonfiglio 2019 | 3 | 2 | 2 | 7 |
| Wang 2019 | 3 | 2 | 2 | 7 |
| Barca 2020 | 3 | 2 | 3 | 8 |
| Hong 2020 | 3 | 2 | 3 | 8 |
| McKay 2020 | 3 | 2 | 3 | 8 |
| Ng 2020 | 3 | 2 | 2 | 7 |
| Christou 2021 | 3 | 2 | 3 | 8 |
| Kaderli 2021 | 3 | 2 | 3 | 8 |
| Liu 2021 | 3 | 2 | 3 | 8 |
| Chatziralli 2022 | 3 | 2 | 3 | 8 |
| D'Aloisio 2022 | 3 | 2 | 3 | 8 |
